# A Major Facilitator Superfamily Transporter Regulated by the Stress-Responsive Transcription Factor Yap1 Is Required for Resistance to Fungicides, Xenobiotics, and Oxidants and Full Virulence in *Alternaria alternata*

**DOI:** 10.3389/fmicb.2018.02229

**Published:** 2018-09-18

**Authors:** Hsien-Che Lin, Pei-Ling Yu, Li-Hung Chen, Hsieh-Chin Tsai, Kuang-Ren Chung

**Affiliations:** Department of Plant Pathology, College of Agriculture and Natural Resources, National Chung-Hsing University, Taichung, Taiwan

**Keywords:** citrus, efflux, reactive oxygen species, pathogen, virulence

## Abstract

*Alternaria alternata* relies on the ability to produce a host-selective toxin and to detoxify reactive oxygen species to successfully colonize the host. An *A. alternata* major facilitator superfamily transporter designated AaMFS54 was functionally characterized by analysis of loss- and gain-of-function mutations to better understand the factors required for fungal pathogenesis. *AaMFS54* was originally identified from a wild-type expression library after being subtracted with that of a mutant impaired for the oxidative stress-responsive transcription regulator Yap1. AaMFS54 contains 14 transmembrane helixes. Fungal mutant lacking *AaMFS54* produced fewer conidia and increased sensitivity to many potent oxidants (potassium superoxide and singlet-oxygen generating compounds), xenobiotics (2,3,5-triiodobenzoic acid and 2-chloro-5-hydroxypyridine), and fungicides (clotrimazole, fludioxonil, vinclozolin, and iprodione). *AaMFS54* mutant induced necrotic lesion sizes similar to those induced by wild-type on leaves of susceptible citrus cultivars after point inoculation with spore suspensions. However, the mutant produced smaller colonies and less fluffy hyphae on the affected leaves. Virulence assays on citrus leaves inoculated by spraying with spores revealed that *AaMFS54* mutant induced less severe lesions than wild-type, indicating the requirement of AaMFS54 in pathogenesis. All defective phenotypes were restored in a strain re-acquiring a functional copy of *AaMFS54*. Northern blotting analysis revealed that the expression of *AaMFS54* was suppressed by xenobiotics. The current studies indicate that the Yap1-mediated transporter plays a role in resistance to toxic oxidants and fungicides in *A. alternata*. The relationships of MFS transporters with other regulatory components conferring stress resistance and *A. alternata* pathogenesis are also discussed.

## Introduction

*Alternaria* species have been documented to cause diseases in more than 400 plant species, including many economically important crops: citrus, apple, rice, strawberry, pear, tomato, broccoli, cauliflower, carrot, potato, tobacco, as well as many ornamental and weed species (Thomma, 2003). *A. alternata* alone can infect nearly 100 plant species and many pathotypes display host specificity due to the ability to produce host selective toxins (HST). The production of HST by *A. alternata* is important for pathogenesis because HST-deficient mutants are unable to induce lesions on host plants (Hatta et al., 2002; Ito et al., 2004). There are two different *A. alternata* pathotypes affecting citrus. The rough lemon pathotype produces ACRL toxin, which is toxic only to lemon (*C. jambhiri* Lush) and Rangpur lime (*C. x limonia* Osbeck). The tangerine pathotype produces ACT toxin, which affects tangerines (*C. reticulate* Blanco), grapefruit (*C. paradise* Macfad.), and their hybrids (Akimitsu et al., 2003).

In addition to HST, *A. alternata* has to overcome toxic reactive oxygen species (ROS) produced by the host plant to successfully colonize and obtain nutrients from the affected tissue. Previous studies have demonstrated that the ability to detoxify ROS mediated by the stress-responsive transcription regulator Yap1, the mitogen-activated protein (MAP) kinase Hog1, and the stress response regulator Skn7 plays a critical role for pathogenesis of *A. alternata* on citrus (Lin et al., 2009; Yang et al., 2009; Lin and Chung, 2010; Chen et al., 2012). Yap1 is a transcriptional regulator containing the basic leucine zipper (bzip) domain and has been demonstrated to be required for resistance to ROS, multidrug, and heavy metals in fungi and yeasts (Alarco and Raymond, 1999; Toone and Jones, 1999; Toone et al., 2001). The tangerine pathotype of *A. alternata* impaired for Yap1, Hog1, or Skn7 increases sensitivity to ROS and multidrug and reduces virulence in citrus, confirming the importance of the ability to detoxify ROS in pathogenesis of *A. alternata*.

Many genes potentially regulated by Yap1 were identified from a cDNA library prepared from the tangerine pathotype after being subtracted with a *Yap1* mutant cDNA. Of them, many genes encode putative transporters including major facilitator superfamily (MFS) and ATP-binding cassette (ABC) transporters (Lin et al., 2011). Fungal transporters play an important role in multidrug and fungicide resistance (Gulshan and Moye-Rowley, 2007; Coleman and Mylonakis, 2009; Wang et al., 2012; dos Santos et al., 2014; Wu et al., 2016). Analysis of loss- and gain-of-function mutations in an *A. alternata* MFS transporter designated AaMFS19 has allowed us to determine its functions to be associated with cellular resistance to oxidative stress and fungicides including clotrimazole, fludioxonil, and kocide (Chen et al., 2017). The expression of *AaMFS19* is regulated by Yap1, Hog1, and Skn7.

To better understand the molecular mechanisms implicated in resistance to ROS and xenobiotics and fungal pathogenesis, the *AaMFS54* encoding a 14-helix MFS transporter was functionally inactivated by targeted gene disruption in the tangerine pathotype of *A. alternata*. The results indicated that AaMFS19 and AaMFS54 transporters had shared and unique functions to minimize the toxicity of oxidative stress-generating chemicals and fungicides. Unlike *AaMFS19*, expression of the *AaMFS54* gene was primarily regulated by Yap1 but not Hog1. The interplays among different regulators and proteins leading to stress resistance and *A. alternata* pathogenesis were also discussed.

## Materials and Methods

### Fungal Strains and Maintenance

The wild-type EV-MIL31 isolate of *Alternaria alternata* was isolated from a diseased Minneola tangelo (Lin et al., 2009) and used for genetic modifications. Fungal isolates mutated at *Yap1* or *Hog1* were generated in previous studies (Lin et al., 2009; Lin and Chung, 2010). The YCp strain was identified from a Yap1 null mutant after being transformed a functional copy of *Yap1*. Fungal isolates were cultured on potato dextrose agar (PDA) (Difco, Sparks, MD, United States) with a layer of sterile cellophane when DNA or RNA purification was needed. For preparation of fungal protoplasts, fungal isolates were cultured in 50 ml potato dextrose broth (PDB) for 4 to 5 days. For conidial formation, fungal isolates were cultured on PDA plates. The plates placed in a plastic box were incubated under fluorescent light for 4 to 5 days. After transformation, fungal strains were recovered from regeneration medium supplemented with 3.42 g/ml sucrose as an osmotic reagent (Chung et al., 2002).

### Targeted Gene Replacement

**FIGURE 1:**
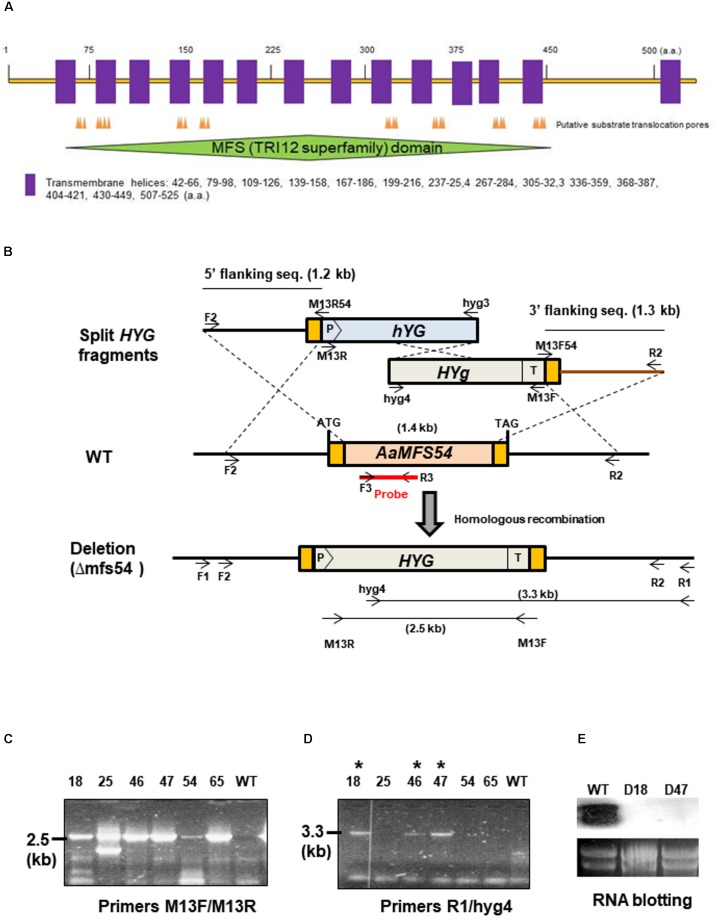
Targeted disruption of *AaMFS54* in the tangerine pathotype of *Alternaria alternata* using a split marker approach. **(A)** Functional domains and transmembrane helices found in the AaMFS54. **(B)** Schematic illustration of generation of truncated but overlapping hygromycin phosphotransferase gene (*HYG*) under control by the *Aspergillus nidulans* *trpC* promoter (P) and terminator (T) within *AaMFS54*. Oligonucleotide primers used to amplify *AaMFS54* fragments and to label the probe are indicated. **(C)** Amplification of DNA fragments from genomic DNA of wild type (WT) and transformants with the primer M13F and M13R. **(D)** Amplification of DNA fragments from genomic DNA of wild type (WT) and transformants with the primer R2 paired with hyg4, indicating that *AaMFS54* is deleted in transformants D18, D46, and D47 (indicated by ^∗^). **(E)** Northern blot hybridization of fungal RNA with an *AaMFS54*-specific probe reveals the lack of the *AaMFS54* gene transcripts in both D18 and D47 strains.

A cDNA clone #54 (accession no. OWY53006.1) encoding an *A. alternata* major facilitator superfamily (AaMFS) was identified from a wild-type cDNA library after being subtracted with that of a *Yap1* null mutant (Lin et al., 2011). *AaMFS54* open reading frame (ORF) and its flanking sequence were obtained from the completed genome sequence of *A. alternata* (Wang et al., 2016). Fungal genomic DNA was isolated using a DNeasy Plant kit (Qiagen, Valencia, CA). A modified split marker approach (Chung and Lee, 2014) was used to delete genes in *A. alternata* by a bacterial phosphotransferase B gene (*HYG*) cassette under the control of the *Aspergillus nidulans* *trpC* gene promoter and terminator, conferring resistance to hygromycin as a selectable marker. To disrupt *AaMFS54*, shortened but partially overlapping *HYG* fragments M13R/hyg3 and hyg4/M13F were obtained by PCR and joined with 5′ and 3′ truncated *AaMFS54* fragments (F2-M13R54 and M13F54-R2), respectively, by second-round PCR (**Figure 1**). DNA fragments were combined (1:1, v/v) and transformed into protoplasts prepared from wild-type using a CaCl_2_ and polyethylene glycol-mediated method as previously described (Chung et al., 2002). Fungal transformants were recovered from a regeneration medium amended with 250 μg/ml hygromycin (Roche Applied Science, Indianapolis, IN) and examined by PCR with hyg3/hyg4 primers and an *AaMFS54*-specific primer (R1) and hyg4 as indicated. The R1 primer whose sequence is not present in the split marker fragments was paired with the hyg4 primer to confirm for the occurrence of homologous integration within *AaMFS54*. This primer set would amplify a fragment only from fungal strains carrying the integration of *HYG* within *AaMFS54*. An expected 2.5-kb fragment was amplified with the primers hyg3 and hyg4 from genomic DNA prepared from six transformants, and no product was amplified from that of wild-type. An expected 3.3-kb fragment was amplified with the primers R1 and hyg4 from genomic DNA of three transformants (D18, D46, and D47), indicative of the successful deletion of *AaMFS54*. Disruption of *AaMFS54* was further validated by Northern blot analysis (**Figure 1**). Oligonucleotide primers used for PCR amplification are listed in **Supplementary Table S1**.

### Genetic Complementation

Full-length *AaMFS54* ORF and its promoter region were amplified with primers F1 and R1 and used for genetic complementation. The *AaMFS54* fragment was co-transformed with the pBARKS1 carrying the *BAR* gene responsible for bialaphos resistance under control of the *Aspergillus nidulans* *trp*C promoter (Pall and Brunelli, 1993) into protoplasts prepared from the D18 mutant. Transformants were recovered from medium amended with 350 μg/ml bialaphos (Phytotechnology Lab., Lenexa, KS). Because fungal strains with *AaMFS54* deficiency increased sensitivity to 0.5 μg/ml fludioxonil fungicide (see below for details), transformants were tested for restoration of cellular resistance to this fungicide.

### Chemical Sensitivity Assays

Sensitivity assays to chemicals were carried out by transferring fungal mycelia as a toothpick point inoculation on PDA containing test compounds and incubated at 28°C. Colony radius was measured at 3 to 9 days. Each treatment contained four replicates, and all experiments were performed at least three times. The difference of radial growth of the disruption mutants was determined in relation to the wild-type grown on the same plate. Percentage change was calculated by dividing the relative difference of the growth by the wild-type growth and multiplying by 100.

### Gene Expression

Northern-blot hybridization was conducted to examine the expression of *AaMFS54* in the wild-type strain grown on PDA covered with a layer of cellophane for 3 days. Mycelium was harvested from medium treated with or without chemicals and used for RNA purification using a TriZol reagent (Invitrogen, Carlsbad, CA, United States). For Northern blot analysis, RNA was electrophoresed and denatured in a formaldehyde-containing agarose gel, blotted onto a nylon membrane, and hybridized to an *AaMFS54* DNA probe according to the procedures described by Sambrook and Russell (2001). The probe was simultaneously amplified and labeled with a digoxigenin (DIG)-11-dUTP by PCR with the *AaMFS54* gene-specific primers F3 and R3 (**Supplementary Table S1**). The probe (**Figure 1**) was identified by an immunological assay using disodium 3-(4-methoxyspiro [1,2-dioxetane-3,2′-(5′-chloro)tricyclo decan]-4-yl)phenyl phosphate (CSPD) as a chemofluorescent substrate for alkaline phosphatase according to the manufacturer’s recommendation (Roche Life Science, Penzberg, Germany). The first strand cDNA was synthesized by an M-MLV reverse transcriptase (Epicentre/Lucigen, Middleton, WI, United States) and used as a template for PCR amplification with gene-specific primers. PCR products were separated by a 1% agarose gel.

### Virulence Assays

Fungal virulence was performed on excised calamondin (*Citrus mitis* Blanco) or Murcott (*Citrus reticulata* Blanco) leaves. Citrus leaves were inoculated by placing 5 μl of conidial suspension (10^4^ conidia per ml) on each spot or sprayed to run-off with conidial suspensions using a min-sprayer. The inoculated leaves were kept in a plastic box for lesion formation. Each fungal strain was tested on at least five leaves, and experiments were repeated three times.

### Statistical Analysis

The significance of treatments was determined by analysis of variance and means separated by student *t*-test (*P* < 0.05).

## Results

### Identification and Characterization of the MFS-Coding Gene

The *A. alternata* *AaMFS54* gene was previously identified from the wild-type cDNA pool after being subtracted with that of a *Yap1* mutant and was found to encode a MFS transporter. Sequence analysis revealed that *AaMFS54* has a 1,614-bp open reading frame disrupted by four small introns that would encode a polypeptide of 538 amino acids after translation. AaMFS54 was found to contain several substrate translocation pores (**Figure 1A**). Hydropathy analysis revealed that AaMFS54 has 14 putative transmembrane domains. A MFS domain belonging to the fungal trichothecene efflux pump (TRI12) superfamily was also found in AaMFS54.

### Targeted Gene Disruption

**FIGURE 2:**
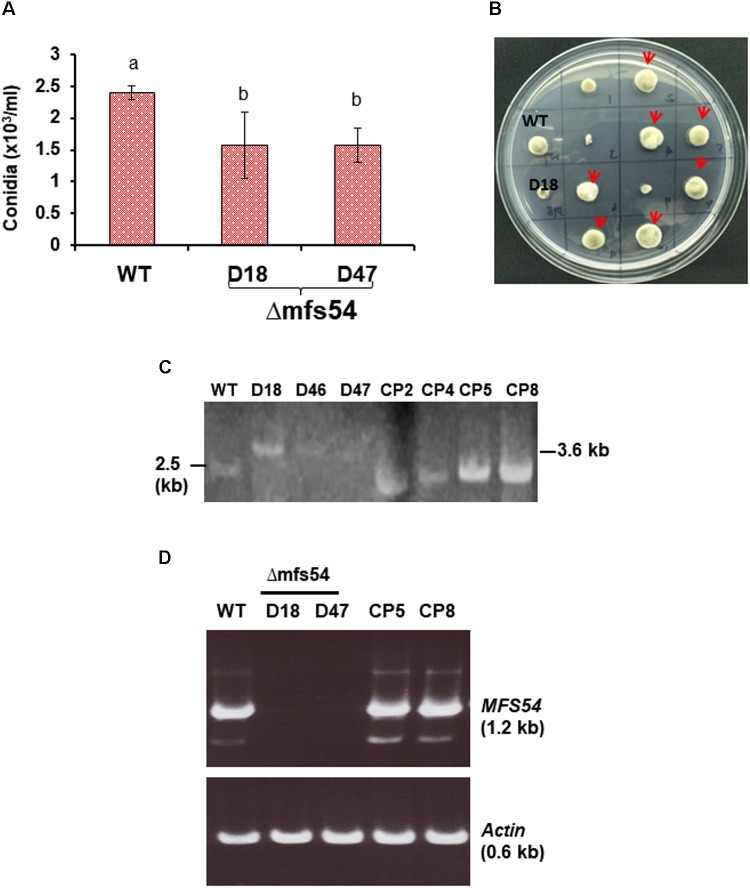
Phenotypic characterization of *AaMFS54* strains. **(A)** Production of conidia by the wild-type (WT) and two *AaMFS54* impaired mutants (Δmfs54-D18 and Δmfs54-D47) of *A. alternata* grown on PDA. Means followed by the same letters were not significantly different according to student *t*-test (*P* ≤ 0.05). **(B)** Growth of WT, D18 and fungal transformants from D18 protoplasts after being co-transformed with a copy of *AaMFS54* and pBarks1 plasmid on PDA amended with 0.5 μg/ml fludioxonil 7 days. Several transformants (indicated by arrows) displayed WT growth, indicative of expression of *AaMFS54*. **(C)** Image of DNA fragments amplified from genomic DNA of WT, three Δmfs54 mutants (D18, D46, and D47) and four complementation strains (CP2, CP4, CP5, and CP8) with primers SSH54P and SSH54T. **(D)** Reverse transcriptase-PCR products from amplified from RNA samples isolated from *A. alternata* strains with SSH54QF1 and SSH54QR2 primers (top panel) and actin gene primers (bottom panel), indicating the expression of *AaMFS54* in WT, CP5, and CP8. No product was amplified from RNA samples prepared from D18 and D47.

To investigate the functions of AaMFS54 transporter, a split marker approach was employed to delete *AaMFS54* in the wild-type strain of *A. alternata*. Introduction of two truncated but overlapping *HYG* gene cassette fusing with 5′ or 3′ *AaMFS54* fragment (**Figure 1B**) resulted in strains conferring resistance to hygromycin. Of six transformants recovered from hygromycin-containing medium, three had an expected deletion of *AaMFS54* as evidenced by diagnostic PCR with a *Hyg* primer pairing with an *AaMFS54* primer whose sequences were not present in the split marker fragments (**Figures 1C,D**). Only transformants carrying a successful integration of *HYG* within *AaMFS54* would yield a product with the primer pairing with a *HYG* gene primer. Two mutant strains (designated Δ*mfs54* D18 and Δ*mfs54* D47) were used for further analyses. Northern blot hybridization (**Figure 1E**) of total RNA prepared from D18 and D47 with an *AaMFGS54*-specific probe failed to detect an expected *AaMFS54* transcript as the wild-type, confirming that both D18 and D47 were *AaMFS54* null mutants. Δ*mfs54* D18 and Δ*mfs54* D47 produced fewer conidia than wild-type (**Figure 2A**). Several transformants showing an increased resistance to 0.5 μg/ml fludioxonil were identified after transforming a copy of *AaMFS54* into protoplasts prepared from Δ*mfs54*-D18 (**Figure 2B**). PCR and RT-PCR analyses using *AaMFS54*-specific primers also confirmed successful complementation seen in recovered transformants (**Figures 2C,D**).

### AaMFS54 Transporter Confers Resistance to Different Chemicals

**FIGURE 3:**
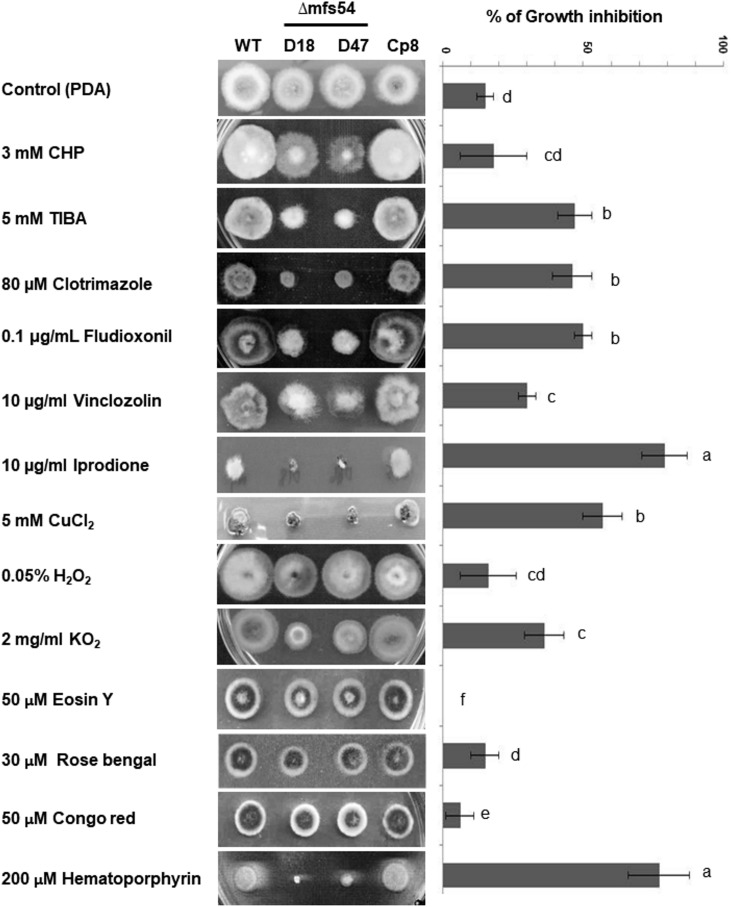
The *Alternaria alternata* major facilitator superfamily transporter (AaMFS54) is required for resistance to xenobiotics and fungicides. Image of the wild-type (WT), two Δmfs54 deletion mutants (D18 and D47), and the CP8 complementation strain grown on potato dextrose agar (PDA) amended with indicated chemicals (left panel). Only representatives are shown. Quantitative analysis of chemical sensitivity is shown on the right panel. Growth reduction was calculated as a cumulative percentage of growth of wild-type and Δmfs54 strains grown on the same plate. The data presented are the mean and standard deviation of two independent experiments with four replicates. Abbreviations: CHP, 2-chloro-5-hydroxypyridine; TIBA, 2,3,5-triiodobenzoic acid. Means followed by the same letters were not significantly different according to student *t*-test (*P* ≤ 0.05).

Compared to wild-type, Δ*mfs54* reduced radial growth by ∼20% on potato dextrose agar plate. Δ*mfs54* increased sensitivity to xenobiotics [2,3,5-triiodobenzoic acid (TIBA) and 2-chloro-5-hydroxypyridine (CHP)], CuCl_2_ and several fungicides including clotrimazole, fludioxonil, vinclozolin, and iprodione (**Figure 3**). Although Δ*mfs54* colony diameter was not obviously different from untreated control on medium amended with CHP, the mutant strains apparently produced thinner colonies. Δ*mfs54* was more sensitive to potassium superoxide (KO_2_) and singlet oxygen-generating compound hematoporphyrin than wild-type. However, Δ*mfs54* showed no increased sensitivity on medium containing H_2_O_2_, singlet oxygen-generating compounds (rose Bengal and eosin Y), or a cell wall disturbing compound Congo red. Transformation of a functional copy of *AaMFS54* into protoplasts prepared from Δ*mfs54*-D18 yielded a Cp8 strain, which displayed wild-type resistance to all test chemicals.

### AaMFS54 Is Required for Fungal Virulence

**FIGURE 4:**
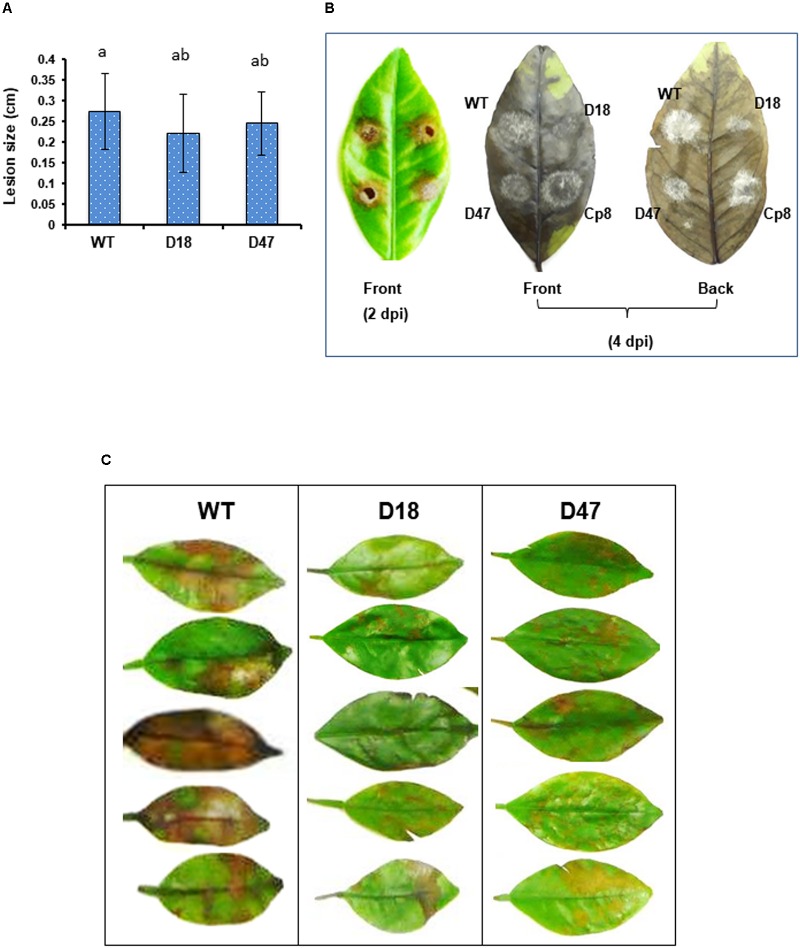
The *A. alternata* major facilitator superfamily transporter (AaMFS54) is a virulence determinant. **(A)** Conidial suspensions (10^4^ conidia per ml) prepared from WT and Δmfs54 strains were inoculated by dropping 5 μl on detached calamondin leaves (*n* = 8). The diameter of lesions was measured 2 days post inoculation (dpi). Means followed by the same letters were not significantly different according to student *t*-test (*P* ≤ 0.05). **(B)** Formation of necrotic lesions (2 dpi) and fungal colonies and fluffy hyphae on necrotic lesions (4 dpi). **(C)** Development of necrotic lesions on detached calamondin leaves inoculated by spraying with conidial suspensions 2 dpi. The mock controls were treated with water only. The leaves were maintained in a moisture chamber for lesion development. Only representatives are shown.

Virulence assays performed on detached calamondin or Murcott leaves inoculated by placing conidial suspensions on the leaves revealed that Δ*mfs54* induced necrotic lesions comparable to wild-type (**Figure 4A**). However, both Δ*mfs54* strains produced much smaller colonies and less fluffy hyphae than wild-type and Cp8 strain on affected leaves 4 days post inoculation (**Figure 4B**). To mimic natural infection, virulence assays were also conducted on calamondin leaves inoculated by uniformly spraying with conidial suspensions. The results revealed that Δ*mfs54* induced less severe lesions than wild-type (**Figure 4C**).

### Differential Expression of AaMFS54

**FIGURE 5:**
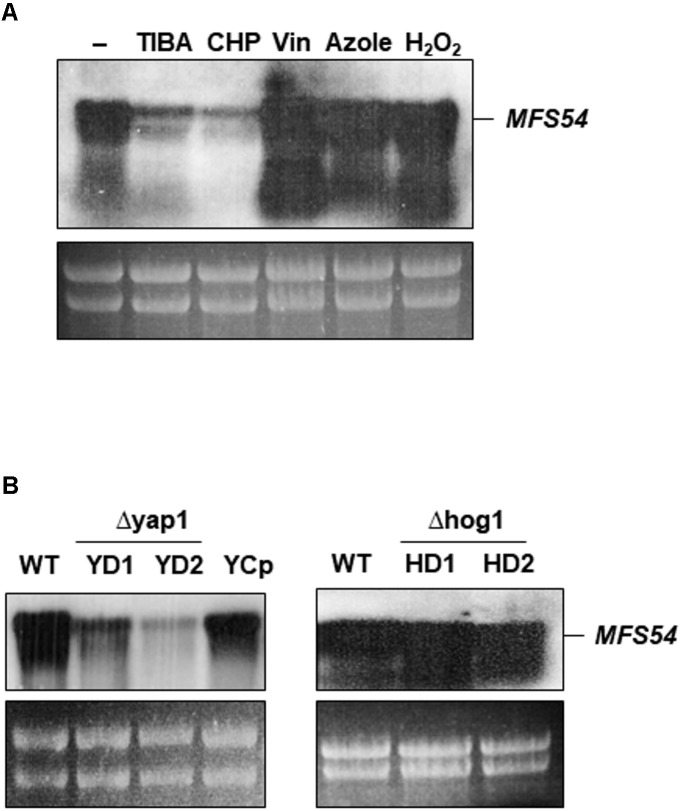
Expression of the *AaMFS54* gene by Northern blot analysis. **(A)** RNA was purified from wild-type (WT) grown on PDA with or without chemicals. Abbreviations: TIBA, 2,3,5-triiodobenzoic acid; CHP, 2-chloro-5-hydroxypyridine; Vin, vinclozolin; and Azole, clotrimazole. **(B)** Detection of the *AaMFS54* transcript in WT, two Δyap1 strains (YD1 and YD2) impaired at the Yap1 transcription regulator, the YCp strain re-acquiring a functional *Yap1* gene, and two Δhog1 strains (HD1 and HD2) impaired at the Hog1 MAP kinase. RNA was electrophoresed on a formaldehyde-containing gel, blotted to membrane, and probed with an *AaMFS54*-specific probe (**Figure 1B**). Ribosomal RNA after being stained with ethidium bromide is used to indicate the loading of the samples.

Northern blotting revealed that the expression of *AaMFS54* was down-regulated in the wild-type grown on a medium containing TIBA or CHP (**Figure 5A**). The expression of *AaMFS54* was induced in the wild-type grown on medium amended with H_2_O_2_, clotrimazole, or vinclozolin. Northern blotting revealed further that the accumulation of the *AaMFS54* transcript decreased in fungal strains defective for the stress-responsive transcription regulator Yap1 (**Figure 5B**). The level of the *AaMFS54* transcript in the complementation strain YCp expressing a functional copy of *Yap1* was similar to that of the wild-type. However, the expression of *AaMFS54* was not affected in two fungal strains with the defective Hog1 MAP kinase.

## Discussion

Fungal transporters are involved in cellular resistance to numerous chemicals, metal ions, molecules, and drugs. Investigation of *A. alternata* molecular determinants of H_2_O_2_ resistance has allowed us to identify several genes coding for membrane transporters (Lin et al., 2011). We have previously characterized an *A. alternata* major facilitator superfamily transporter 19 (AaMFS19) to be required for resistance to a wide range of oxidants, fungicides, and xenobiotics (Chen et al., 2017). AaMFS19, containing 12 transmembrane domains, also plays a role in fungal virulence. In the present study, we report functional characterization of second MFS transporter designated AaMFS54 to determine its role in cellular resistance to various chemicals, fungicides, and in fungal pathogenesis.

AaMFS54 protein contains 14 transmembrane domains. Phenotypic analysis of fungal mutants carrying an *AaMFS54* deficiency reveals that AaMFS54 is required for resistance to a great diversity of chemicals, including 2,3,5-triiodobenzoic acid (TIBA) and 2-chloro-5-hydroxypyridine (CHP), clotrimazole, fludioxonil, vinclozolin, iprodione, CuCl_2_, KO_2_, and hematoporphyrin. Previous studies have shown that fungal mutant strains defective for AaMFS19 containing 12 transmembrane domains are also hypersensitive to those fungicides and chemicals (Chen et al., 2017). CHP is a potent oxidant because it could react with H_2_O_2_ to produce superoxide and hydroxyl radicals in the presence of Cu^2+^ (Watanabe et al., 1998; Nerud et al., 2001). Both CHP and TIBA have been demonstrated to be toxic to *A. alternata*, suppressing gene expression, causing the formation of shorter hyphal branches, reducing growth, and suppressing conidial formation and germination (Chen et al., 2017). Both AaMFS19 and AaMFS54 are involved in resistance to CHP and TIBA, even though expression of the two coding genes is down-regulated in *A. alternata* strain treated with CHP or TIBA. The *A. alternata* mutants carrying AaMFS19 or AaMFS54 deficiency display increased sensitivity to fludioxonil, vinclozolin, iprodione, and clotrimazole fungicides, confirming further the important role of MFS transporters in multidrug resistance and stress adaptation in fungi (Del Sorbo et al., 2000; Hayashi et al., 2002; Roohparvar et al., 2007, 2008; Costa et al., 2014; Paul and Moye-Rowley, 2014; Xu et al., 2014; Redhu et al., 2016).

Both AaMFS19 and AaMFS54 coding sequences were originally identified from a cDNA library prepared from the wild-type strain of *A. alternata* after subtraction with cDNA prepared from a *Yap1* mutant (Lin et al., 2011). Northern blot hybridization confirms that accumulation of the *AaMFS19* and *AaMFS54* transcripts is coordinately regulated by the stress responsive transcription factor Yap1 (Chen et al., 2017 and this study). However, expression of *AaMFS19* but not *AaMFS54* is regulated by the stress-related regulators Hog1 and Skn7, supporting that AaMFS19 and AaMFS54 have divergent functions. Moreover, because Yap1, Hog1, and Skn7 regulators have been demonstrated to play a critical role in resistance to oxidative stress (Chung, 2014), it appears that ROS resistance in *A. alternata* is, at least in part, mediated by membrane-bound transporters. However, it is unlikely that MFS transporters are specifically functioning at ROS because functional analysis reveals that AaMFS19 and AaMFS54 are not required for resistance to H_2_O_2_ or many ROS-generating oxidants. For example: *AaMFS54* mutant strains are hypersensitive to hematoporphyrin, but not eosin Y and rose Bengal, even though all could react with O_2_ to produce toxic singlet oxygen upon exposure to light (Daub et al., 2013). *AaMFS54* mutant strains also display wild-type sensitivity to singlet oxygen-generating compounds, toluidine blue (200 μM), methylene blue (200 μM), cercosporin (50 μM), and elsinochromes (30 μM) (data not shown). In contrast, *AaMFS19* mutant strains increased sensitivity to eosin Y, rose Bengal, hematoporphyrin, methylene blue, and cercosporin. The results clearly indicate that AaMFS19 and AaMFS54 transporters have shared and unique substrate preferences and functions to mitigate the toxicity of oxidative stress-generating chemicals. Expression of the MFS transporter coding genes in the budding yeast *Saccharomyces cerevisiae* is known to be regulated by Yap1 and other stress-related transcription factors (dos Santos et al., 2014). MFS transporters could function directly to avoid the toxicity of chemicals by pumping them out of the cell. MFS transporters could also act indirectly against toxic chemicals or oxidants by altering metabolic regulation or by changing the plasma membrane compositions (Coleman and Mylonakis, 2009; Dhaoui et al., 2011; Krüger et al., 2013; Rios et al., 2013).

Pathogenicity tests conducted on detached calamondin or Murcott leaves inoculated by placing conidial suspensions (5 μl, 10^4^ conidia per ml) reveal no significant differences in lesion diameters induced by the wild-type and the *AaMFS54* mutant. The *AaMFS54* mutant reduced growth and produced much smaller colonies and less fluffy hyphae than wild-type. Pathogenicity assays on citrus leaves inoculated by spraying with conidial suspensions (mimicking natural infection) reveal that the *AaMFS54* mutant induced less severe lesions than wild-type. Similar results were also observed in the fungal mutant impaired for AaMFS19 (Chen et al., 2017). The discrepancy between point and spray inoculations is likely due to the concentrations of conidia present in the infection court.

**FIGURE 6:**
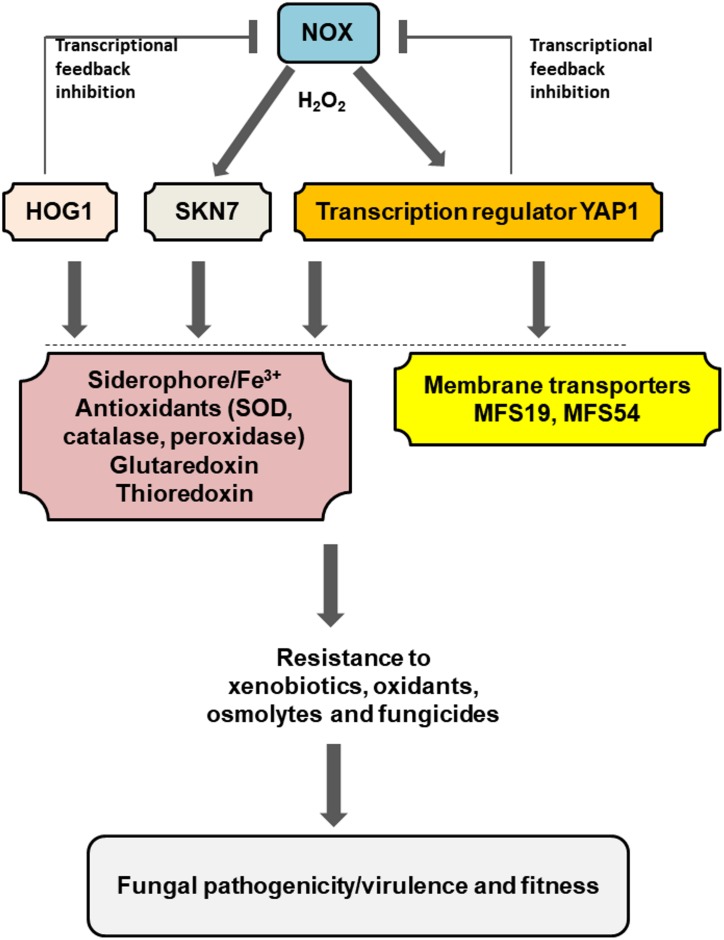
Schematic illustration of a regulation network associated with pathogenesis and fitness in the tangerine pathotype of *A. alternata*. Low level of H_2_O_2_ generated by the NADPH oxidases (NOX) plays a central role in the activation of Yap1, Hog1, and Skn7, which subsequently regulate the expression of numerous genes associated with iron uptake and resistance to different stress including oxidants xenobiotics, fungicides, and osmolytes. Two MFS transporters regulated by Yap1 and/or Hog1 play an important role in the process. Transcriptional feedback inhibition of the Nox-coding genes by Yap1 and Hog1 was also observed. Detailed description can be found in the text.

Current and previous studies have begun to shed a light on the interplays among different regulators and proteins required for stress resistance and fungal pathogenicity/virulence of *A. alternata* (**Figure 6**). A low level of H_2_O_2_ generated by the NADPH oxidase (Nox) likely activates Yap1, Hog1, and Skn7 at transcriptional and/or post-translational levels, which could subsequently enhance siderophore biosynthesis foriron uptake and storage and promote antioxidant activities including superoxide dismutase (SOD), catalase, and peroxidase,all leading to oxidative stress resistance (Chen et al., 2013, 2014). Yap1, Hog1, and Skn7 could also activate gluataredoxin and thioredoxin systems to cope with oxidative stress (Yang et al., 2016; Ma et al., 2018). Because H_2_O_2_ is toxic to cells, Yap1, Hog1, and Skn7 would suppress the expression of the *Nox* genes (transcriptional feedback inhibition) to avoid the unrestraint production of H_2_O_2_. In addition to oxidative stress, Yap1, Hog1, and Skn7 are involved in the adaptation to osmolytes, xenobiotics, or fungicides. These functions are in part mediated by the membrane-bound MFS transporters. All of these components would allow *A. alternata* to deal with environmental stress and enhance its ability to colonize the host plant.

## Author Contributions

K-RC designed the experiments and wrote the main manuscript text. H-CL, P-LY, L-HC, and H-CT performed the experiments and acquired the data. H-CL, P-LY, L-HC, H-CT, and K-RC analyzed and interpreted the data. All authors reviewed the manuscript.

## Conflict of Interest Statement

The authors declare that the research was conducted in the absence of any commercial or financial relationships that could be construed as a potential conflict of interest.

## References

[B1] Akimitsu K. Peever T. L. Timmer L. W. (2003). Molecular, ecological and evolutionary approaches to understanding *Alternaria* diseases of citrus. *Mol. Plant Pathol.* 4 435–446. 10.1046/j.1364-3703.2003.00189.x 20569403

[B2] Alarco A. Raymond M. (1999). The bZip transcription factor Cap1p is involved in multidrug resistance and oxidative stress response. *J. Bacteriol.* 181 700–708. 9922230 10.1128/jb.181.3.700-708.1999 PMC93433

[B3] Chen L. H. Lin C. H. Chung K. R. (2012). Roles for SKN7 response regulator in stress resistance, conidiation and virulence in the citrus pathogen *Alternaria alternata*. *Fungal Genet. Biol.* 49 802–813. 10.1016/j.fgb.2012.07.006 22902811

[B4] Chen L. H. Lin C. H. Chung K. R. (2013). A nonribosomal peptide synthetase mediates siderophore production and virulence in the citrus fungal pathogen *Alternaria alternata*. *Mol. Plant Pathol.* 14 497–505. 10.1111/mpp.12021 23438010 PMC6638914

[B5] Chen L. H. Tsai H. C. Chung K. R. (2017). A major facilitator superfamily transporter-mediated resistance to oxidative stress and fungicides requires Yap1, Skn7, and MAP kinases in the citrus fungal pathogen *Alternaria alternata*. *PLoS One* 12:e0169103. 10.1371/journal.pone.0169103 28060864 PMC5218470

[B6] Chen L. H. Yang S. L. Chung K. R. (2014). Resistance to oxidative stress via regulating siderophore-mediated iron-acquisition by the citrus fungal pathogen *Alternaria alternata*. *Microbiology* 160 970–979. 10.1099/mic.0.076182-0 24586035

[B7] Chung K. R. (2014). Reactive oxygen species in the citrus fungal pathogen *Alternaria alternata*: the roles of NADPH oxidase. *Physiol. Mol. Plant Pathol.* 88 10–17. 10.1016/j.pmpp.2014.08.001

[B8] Chung K. R. Lee M. H. (2014). “Split marker-mediated transformation and targeted gene disruption in filamentous fungi,” in *Genetic Transformation Systems in Fungi* Vol. 2 eds van den Berg M. A. Maruthachalam K. (Berlin: Springer International Publishing), 175–180.

[B9] Chung K. R. Shilts T. Li W. Timmer L. W. (2002). Engineering a genetic transformation system for *Colletotrichum acutatum*, the causal fungus of lime anthracnose and postbloom fruit drop. *FEMS Microbiol. Lett.* 213 33–39. 10.1111/j.1574-6968.2002.tb11282.x 12127485

[B10] Coleman J. J. Mylonakis E. (2009). Efflux in fungi: la Pièce de Résistance. *PLoS Pathog.* 5:e1000486. 10.1371/journal.ppat.1000486 19557154 PMC2695561

[B11] Costa C. Dias P. J. Sá-Correia I. Teixeira M. C. (2014). MFS multidrug transporters in pathogenic fungi: do they have real clinical impact? *Front. Physiol.* 5:197. 10.3389/fphys.2014.00197 24904431 PMC4035561

[B12] Daub M. E. Herrero S. Chung K. R. (2013). Reactive oxygen species in plant pathogenesis: the role of perylenequinone photosensitizers. *Antioxid. Redox Signal.* 19 970–989. 10.1089/ars.2012.5080 23259634

[B13] Del Sorbo G. Schoonbeek H. De Waard M. A. (2000). Fungal transporters involved in efflux of natural toxic compounds and fungicides. *Fungal Genet. Biol.* 30 1–15. 10.1006/fgbi.2000.1206 10955904

[B14] Dhaoui M. Auchere F. Blaiseau P. L. Lesuisse E. Landoulsi A. Camadro J. M. (2011). Gex1 is a yeast glutathione exchanger that interferes with pH and redox homeostasis. *Mol. Biol. Cell* 22 2054–2067. 10.1091/mbc.E10-11-0906 21490148 PMC3113770

[B15] dos Santos S. C. Teixeira M. C. Dias P. J. Sá-Correia I. (2014). MFS transporter required for multidrug/multixenobiotic (MD/MX) resistance in the model yeast: understanding their physiological function through post-genomic approaches. *Front. Physiol.* 5:180 10.3389/fphys.2014.00180 PMC4021133 24847282

[B16] Gulshan K. Moye-Rowley W. S. (2007). Multidrug resistance in fungi. *Eukaryot. Cell* 6 1933–1942. 10.1128/EC.00254-07 17873085 PMC2168405

[B17] Hatta R. Ito K. Hosaki Y. Tanaka T. Tanaka A. Yamamoto M. (2002). The conditionally dispensable chromosome controls host-specific pathogenicity in the fungal plant pathogen *Alternaria alternata*. *Genetics* 161 59–70. 12019223 10.1093/genetics/161.1.59 PMC1462115

[B18] Hayashi K. Schoonbeek H. De Waard M. A. (2002). Bcmfs1, a novel major facilitator superfamily transporter from *Botrytis cinerea*, provides tolerance towards the natural toxic compounds camptothecin and cercosporin and towards fungicides. *Appl. Environ. Microbiol.* 68 4996–5004. 10.1128/AEM.68.10.4996-5004.2002 12324349 PMC126426

[B19] Ito K. Tanaka T. Hatta R. Yamamoto M. Akimitsu K. Tsuge T. (2004). Dissection of the host range of the fungal plant pathogen *Alternaria alternata* by modification of secondary metabolism. *Mol. Microbiol.* 52 399–411. 10.1111/j.1365-2958.2004.04004.x 15066029

[B20] Krüger A. Vowinckel J. Mulleder M. Grote P. Capuano F. Bluemlein K. (2013). Tpo1-mediated spermine and spermidine export controls cell cycle delay and times antioxidant protein expression during oxidative stress response. *EMBO Rep.* 14 1113–1119. 10.1038/embor.2013.165 24136413 PMC3981086

[B21] Lin C. H. Chung K. R. (2010). Specialized and shared functions of the histidine kinase- and HOG1 MAP kinase-mediated signaling pathways in *Alternaria alternata*, a filamentous fungal pathogen of citrus. *Fungal Genet. Biol.* 47 818–827. 10.1016/j.fgb.2010.06.009 20601043

[B22] Lin C. H. Yang S. L. Chung K. R. (2009). The YAP1 homolog-mediated oxidative stress tolerance is crucial for pathogenicity of the necrotrophic fungus *Alternaria alternata* in citrus. *Mol. Plant Microbe Interact.* 22 942–952. 10.1094/MPMI-22-8-0942 19589070

[B23] Lin C. H. Yang S. L. Chung K. R. (2011). Cellular responses required for oxidative stress tolerance, colonization and lesion formation by the necrotrophic fungus *Alternaria alternata* in citrus. *Curr. Microbiol.* 62 807–815. 10.1007/s00284-010-9795-y 20978890

[B24] Ma H. Wang M. Gai Y. Fu H. Ruan R. Chung K. R. (2018). Thioredoxin and glutaredoxin systems required for oxidative stress resistance, fungicide sensitivity and virulence of *Alternaria alternata*. *Appl. Environ. Microbiol.* 84:e00086-18. 10.1128/AEM.00086-18 29752269 PMC6029089

[B25] Nerud F. Baldrian P. Gabriel J. Ogbeifun D. (2001). Decolorization of synthetic dyes by the fenton reagent and the Cu/pyridine/H2O2 system. *Chemosphere* 44 957–961. 10.1016/S0045-6535(00)00482-3 11513429

[B26] Pall M. L. Brunelli J. P. (1993). A series of six compact fungal transformation vectors containing polylinkers with multiple unique restriction sites. *Fungal Genet. Newslett.* 40 59–62. 10.4148/1941-4765.1413

[B27] Paul S. Moye-Rowley W. S. (2014). Multidrug resistance in fungi: regulation of transporter-encoding gene expression. *Front. Physiol.* 5:143 10.3389/fphys.2014.00143 PMC3997011 24795641

[B28] Redhu A. K. Shah A. H. Prasad R. (2016). MFS transporters of Candida species and their role in clinical drug resistance. *FEMS Yeast Res.* 16:fow043 10.1093/femsyr/fow043 27188885

[B29] Rios G. Cabedo M. Rull B. Yenush L. Serrano R. Mulet J. M. (2013). Role of the yeast multidrug transporter Qdr2 in cation homeostasis and the oxidative stress response. *FEMS Yeast Res.* 13 97–106. 10.1111/1567-1364.12013 23106982

[B30] Roohparvar R. De Waard M. A. Kema G. H. Zwiers L. H. (2007). MgMfs1, a major facilitator superfamily transporter from the fungal wheat pathogen *Mycosphaerella graminicola*, is a strong protectant against natural toxic compounds and fungicides. *Fungal Genet. Biol.* 44 378–388. 10.1016/j.fgb.2006.09.007 17107817

[B31] Roohparvar R. Mehrabi R. Van Nistelrooy J. G. Zwiers L. H. De Waard M. A. (2008). The drug transporter MgMfs1 can modulate sensitivity of field strains of the fungal wheat pathogen *Mycosphaerella graminicola* to the strobilurin fungicide trifloxystrobin. *Pest Manag. Sci.* 64 685–693. 10.1002/ps.1569 18366066

[B32] Sambrook J. Russell D. W. (2001). *Molecular Cloning: A Laboratory Manual*, 3rd Edn New York, NY: Cold Spring Harbor Press.

[B33] Thomma B. P. (2003). *Alternaria* spp: from general saprophyte to specific parasite. *Mol. Plant Pathol.* 4 225–236. 10.1046/j.1364-3703.2003.00173.x 20569383

[B34] Toone W. M. Jones N. (1999). AP-1 transcription factors in yeast. *Curr. Opin. Genet. Dev.* 9 55–61. 10.1016/S0959-437X(99)80008-2 10072349

[B35] Toone W. M. Morgan B. A. Jones N. (2001). Redox control of AP-1-like factors in yeast and beyond. *Oncogene* 20 2336–2346. 10.1038/sj.onc.1204384 11402331

[B36] Wang J. Sun X. Lin L. Zhang T. Ma Z. Li H. (2012). PdMfs1, a major facilitator superfamily transporter from *Penicillium digitatum*, is partially involved in the imazalil-resistance and pathogenicity. *Afr. J. Microbiol. Res.* 6 95–105.

[B37] Wang M. Sun X. Yu D. Xu J. Chung K. R. Li H. (2016). Genomic and transcriptomic analyses of the tangerine pathotype of *Alternaria alternata* in response to oxidative stress. *Sci. Rep.* 6:32437. 10.1038/srep32437 27582273 PMC5007530

[B38] Watanabe T. Koller K. Messner K. (1998). Copper-dependent depolymerisation of lignin in the presence of fungal metabolite, pyridine. *J. Biotechnol.* 62 221–230. 10.1016/S0168-1656(98)00063-7 9729805

[B39] Wu Z. Wang S. Yuan Y. Zhang T. Liu J. Liu D. (2016). A novel major facilitator superfamily transporter in *Penicillium digitatum* (PdMFS2) is required for prochloraz resistance, conidiation and full virulence. *Biotechnol. Lett.* 38 1349–1357. 10.1007/s10529-016-2113-4 27146209

[B40] Xu X. Chen J. Xu H. Li D. (2014). Role of a major facilitator superfamily transporter in adaptation capacity of *Penicillium funiculosum* under extreme acidic stress. *Fungal Genet. Biol.* 69 75–83. 10.1016/j.fgb.2014.06.002 24959657

[B41] Yang S. L. Lin C. H. Chung K. R. (2009). Coordinate control of oxidative stress, vegetative growth and fungal pathogenicity via the AP1-mediated pathway in the rough lemon pathotype of *Alternaria alternata*. *Physiol. Mol. Plant Pathol.* 74 100–110. 10.1016/j.pmpp.2009.09.007

[B42] Yang S. L. Yu P. L. Chung K. R. (2016). The glutathione peroxidase–mediated reactive oxygen species resistance, fungicide sensitivity and cell wall construction in the citrus fungal pathogen *Alternaria alternata*. *Environ. Microbiol.* 18 923–935. 10.1111/1462-2920.13125 26567914

