# Supplementary material for: A Major Facilitator Superfamily Transporter Regulated by the Stress-Responsive Transcription Factor Yap1 Is Required for Resistance to Fungicides, Xenobiotics, and Oxidants and Full Virulence in Alternaria alternata
Source: Front Microbiol. 2018 Sep 18;9:2229. doi: 10.3389/fmicb.2018.02229 (PMC6153361; doi:10.3389/fmicb.2018.02229)
Supplement: Supplementary file 1 [file Table_1.PDF]

**Supplementary Table 1.** Oligonucleotide primers used in this study

| Primer | Sequence (5'-3') | Corresponding gene |
|---|---|---|
| M13R | agcggataacaatttcacacagga | pUCATPH1 |
| M13F | cgccagggttttcccagtcacgac | pUCATPH1 |
| hyg3 | ggatgcctccgctcgaagta | *Hyg*$^{r}$ |
| hyg4 | cgttgcaagacctgcctgaa | *Hyg*$^{r}$ |
| F1 | gcattcgcttcaccaccta | *AaMFS54* forward |
| F2 | atctgcccatgccttggctt | *AaMFS54* forward |
| R1 | caaaggaaggtggagggcat | *AaMFS54* reverse |
| R2 | cgaacgctgaagcccaacta | *AaMFS54* reverse |
| M13F54 | gtcgtgactgggaaaaccctggcgtgcttcagaactcaagagag | *AaMFS54* carrying sequence (underlined) complementary to M13F primer |
| M13R54 | tcctgtgtgaaattgttatccgctagctggattagaaagagctt | *AaMFS54* carrying sequence (underlined) complementary to M13R primer |
| F3 | ttcagtagacagaccaaaat | *AaMFS54* forward (probe labeling) |
| R3 | tgtacagactggaagtaaat | *AaMFS54* reverse (probe labeling) |
| SSH54P | tgagttgcacctgccaacgc | *AaMFS54* forward |
| SSH54T | agtgatcagcagactttgga | *AaMFS54* reverse |
| SSH54QF1 | atatctcacttccatcttcttc | *AaMFS54* forward (RT-PCR) |
| SSH54QR2 | cttgatcatgacactatgaa | *AaMFS54* reverse (RT-PCR) |

pUCATPH1, a plasmid harboring a hygromycin resistance cassette

Hyg$^{r}$, a bacterial phosphotransferase B gene (*HYG*) cassette conferring hygromycin resistance
